# Evaluating the prebiotic effect of oligosaccharides on gut microbiome wellness using in vitro fecal fermentation

**DOI:** 10.1038/s41538-023-00195-1

**Published:** 2023-05-09

**Authors:** Dong Hyeon Lee, Hyunbin Seong, Daniel Chang, Vinod K. Gupta, Jiseung Kim, Seongwon Cheon, Geonhee Kim, Jaeyun Sung, Nam Soo Han

**Affiliations:** 1 Brain Korea 21 Center for Bio-Health Industry, Department of Food Science and Biotechnology, Chungbuk National University, Cheongju, Chungbuk 28644 Republic of Korea; 2 Department of Computer Science and Engineering, University of Minnesota-Twin Cities, Minneapolis, MN 55455 USA; 3 Microbiome Program, Center for Individualized Medicine, Mayo Clinic, Rochester, MN 55905 USA; 4 Division of Surgery Research, Department of Surgery, Mayo Clinic, Rochester, MN 55905 USA; 5 Gaesinbiotech, Cheongju, Chungbuk 28644 Republic of Korea; 6 Division of Rheumatology, Department of Internal Medicine, Mayo Clinic, Rochester, MN 55905 USA

**Keywords:** Metagenomics, Predictive markers

## Abstract

We previously proposed the Gut Microbiome Wellness Index (GMWI), a predictor of disease presence based on a gut microbiome taxonomic profile. As an application of this index for food science research, we applied GMWI as a quantitative tool for measuring the prebiotic effect of oligosaccharides. Mainly, in an in vitro anaerobic batch fermentation system, fructooligosaccharides (FOS), galactooligosaccharides (GOS), xylooligosaccharides (XOS), inulin (IN), and 2’-fucosyllactose (2FL), were mixed separately with fecal samples obtained from healthy adult volunteers. To find out how 24 h prebiotic fermentation influenced the GMWI values in their respective microbial communities, changes in species-level relative abundances were analyzed in the five prebiotics groups, as well as in two control groups (no substrate addition at 0 h and for 24 h). The GMWI of fecal microbiomes treated with any of the five prebiotics (IN (0.48 ± 0.06) > FOS (0.47 ± 0.03) > XOS (0.33 ± 0.02) > GOS (0.26 ± 0.02) > 2FL (0.16 ± 0.06)) were positive, which indicates an increase of relative abundances of microbial species previously found to be associated with a healthy, disease-free state. In contrast, the GMWI of samples without substrate addition for 24 h (–0.60 ± 0.05) reflected a non-healthy, disease-harboring microbiome state. Compared to the original prebiotic index (PI) and α-diversity metrics, GMWI provides a more data-driven, evidence-based indexing system for evaluating the prebiotic effect of food components. This study demonstrates how GMWI can be applied as a novel PI in dietary intervention studies, with wider implications for designing personalized diets based on their impact on gut microbiome wellness.

## Introduction

Diet alters the composition of the gastrointestinal microbiome^1,2^, which in turn has an important role in regulating our overall health^3,4^. Gibson and Roberfroid initially defined “prebiotics” as non-digestible food ingredients that promote the growth and/or activity of one or more bacterial species of the gastrointestinal tract (GIT) in an effort to benefit host health^5^. Recently, this definition has been broadened to “a substrate that is selectively utilized by host microorganisms conferring a health benefit” by the International Scientific Association for Probiotics and Prebiotics (ISAPP)^6^. Traditionally, prebiotics were believed to promote the growth of certain presumed beneficial GIT bacteria, such as *Lactobacillus* and *Bifidobacterium*^5^. In addition to their effects on gut microbiome composition, prebiotics can also produce notable shifts in host metabolic and immune markers. For instance, the intake of non-digestible polysaccharides can lead to reductions in the proinflammatory cytokine IL-6, insulin resistance, and peak post-prandial glucose^7,8^. Furthermore, prebiotics can be selectively utilized by gut bacteria to produce short-chain fatty acids (SCFAs) as metabolic byproducts^9,10^, such as acetate and propionate, which can suppress inflammation and protect the host against pathogenic infections^11,12^. However, despite the potential benefits of prebiotics consumption, a concrete index—derived from data-driven, evidence-based approaches—that can quantitatively evaluate the impact of prebiotics on gut microbiome and host health simultaneously has yet to be demonstrated.

Measuring prebiotic activities of foods on the human gut microbiome, and providing dietary guidelines based on these results, are significant emerging issues in current food science research. To this point, the original prebiotic index (PI) was introduced in 2003 as one of the first metrics to estimate the prebiotic effect of dietary oligosaccharides^13^. This PI equation considers the number of Bifidobacteria, Bacteroides, Lactobacilli, Clostridia, and total bacteria; and assumes that an increase in Bifidobacteria and Lactobacilli populations is a positive effect on health, whereas an increase in Bacteroides and Clostridia is a negative one. (To overcome the limitation that the PI accounts for only four families, a modified PI (PIm) was proposed in 2007^14^ by incorporating the maximum specific growth rates (*μ*_*max*_) of Bifidobacteria, Lactobacilli, Eubacteria, Bacteroides, Clostridia, *Escherichia coli*, and sulfate-reducing bacteria.) However, many *Bacteroides* spp. are no longer simply deemed as harmful, and rather as one of the major commensal bacteria that may positively affect human health^15,16^. Moreover, through the use of next-generation sequencing technology, the genus *Lactobacillus* has recently been divided and reclassified into 25 different genera, including 23 novel genera^17^. For these reasons, PI and PIm can now be viewed as relatively outdated techniques for measuring the prebiotic activities of diets.

We recently introduced the Gut Microbiome Wellness Index (GMWI) (previously called the Gut Microbiome Health Index), which evaluates health status (i.e., likelihood of disease presence independent of the clinical diagnosis) based on a species-level taxonomic profile of a stool sample^18^. The GMWI mathematical formula considers the relative abundances of 50 microbial species found to be associated with human health; more specifically, by examining 4347 human stool metagenomes from 34 published studies, 7 and 43 microbial species were observed more frequently (prevalent) and less frequently (scarce), respectively, in healthy people compared to patients with a disease. Notably, GMWI could distinguish people with or without a disease far better than α-diversity indices (e.g., Shannon Index, species richness), thereby paving the way for noninvasive health monitoring via gut microbiome profiling. In a separate but related matter, we hypothesize that GMWI could be extended to promising translational applications in food science research.

In this study, we explore the proof-of-concept use of GMWI for assessing the prebiotic effect of food ingredients. Mainly, we utilize the following commercial prebiotic oligosaccharides: fructooligosaccharides (FOS), galactooligosaccharides (GOS), xylooligosaccharides (XOS), inulin (IN), and 2’-fucosyllactose (2FL). These oligosaccharides are not only known to reach the large intestine (including distal colon) without being hydrolyzed by human digestive enzymes^19–21^, but also reported to provide prebiotic effects^21–23^. After their incubation with human fecal samples (gut microbiomes) for 24 h in an anaerobic, pH-controlled, in vitro fermentation system, the changes in microbial taxa are analyzed using shotgun metagenomics. Afterward, we calculate GMWI values from relative abundances of the aforementioned Health-prevalent and Health-scarce microbial species related to human health.

## Results

### Microbial taxonomic composition changes

In vitro fecal fermentation of FOS, IN, GOS, XOS, and 2FL, along with subsequent shotgun metagenome analyses, were conducted to evaluate the impact of prebiotics on a microbial community from the human gut. To simulate actual human gut conditions, fecal samples were collected from multiple donors (as a microbiome source), and mixed and incubated inside a pH-controlled anaerobic batch culture system (“Methods”). Experimentation was conducted in triplicates in seven study groups as described below.

**Fig. 1:**
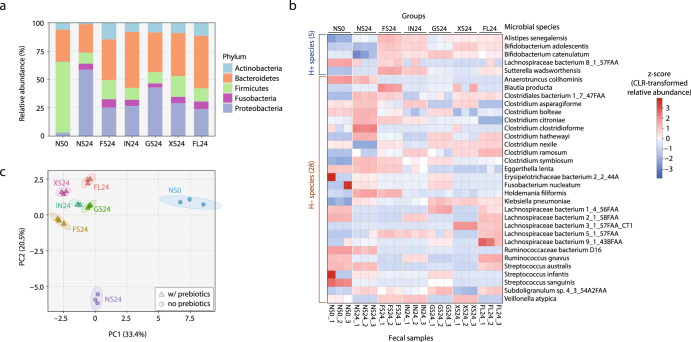
Taxonomic composition of microbial communities after in vitro fecal fermentation of prebiotics for 24 h. **a** Average relative abundances of major phyla in each study group. **b** Relative abundances of 33 Health-prevalent (H+) and Health-scarce (H–) microbial species. Colors on heatmap correspond to z-scores (across all samples) of centered log-ratio (CLR) transformed relative abundances. **c** PCA ordination plot of all samples from the seven study groups. Five prebiotics groups: FS24 fructooligosaccharide fermentation for 24 h, IN24 inulin fermentation for 24 h, GS24 galactooligosaccharide fermentation for 24 h, XS24 xylooligosaccharide fermentation for 24 h, FL24 2’-fucosyllactose fermentation for 24 h. Two control groups: NS0 no substrate addition at 0 h, NS24 no substrate addition for 24 h.

The relative abundances in five main phyla (Actinobacteria, Bacteroidetes, Firmicutes, Fusobacteria, Proteobacteria) after in vitro fecal fermentation are shown in Fig. 1a. For all five prebiotics, the average relative abundances of Actinobacteria, Bacteroidetes, Fusobacteria, and Proteobacteria increased during the 24 h fecal fermentation period, whereas the average relative abundance of Firmicutes largely decreased.

When the microbial taxa changes were analyzed at species level, a total of 236 species were detected (Supplementary Table 1), of which 33 matched the 50 Health-prevalent and Health-scarce species found in our original GMWI study (Fig. 1b and Table 1). Five Health-prevalent species were detected: *Alistipes senegalensis*, *Bifidobacterium adolescentis*, *Bifidobacterium catenulatum*, *Lachnospiraceae bacterium* 8_1_57FAA, and *Sutterella wadsworthensis*. Their abundance levels in the mixed adult feces before fermentation (no substrate at 0 h [NS0]) were generally low (0.00–1.74%). However, for *B. adolescentis* (1.74% in NS0), its relative abundance significantly increased (*P* < 0.05) to 4.73%, 3.99%, 5.59%, and 3.61% after 24 h fermentation of FOS, IN, XOS, and 2FL, respectively. Similarly, all five prebiotics led to an increase in the relative abundance of other Health-prevalent species *B. catenulatum* and *S. wadsworthensis* (*P* < 0.05). On the other hand, 28 total Health-scarce species were detected. The relative abundances of most of these species were observed to have either decreased or stayed in low amounts after 24 h fermentation of the five prebiotics.

**Table 1:** Relative abundances of GMWI Health-prevalent and Health-scarce species in prebiotics-treated and control groups.

Group	Microbial species	NS0	NS24	FS24	IN24	GS24	XS24	FL24
Health-prevalent	*Bifidobacterium catenulatum*	0.03 ± 0.00 ^f^	0.00 ± 0.00 ^g^	0.18 ± 0.00^a^	0.08 ± 0.01^c^	0.17 ± 0.01^b^	0.06 ± 0.00^d^	0.04 ± 0.01^e^
	*Alistipes senegalensis*	0.00 ± 0.00^e^	0.01 ± 0.00^d^	0.04 ± 0.00^a^	0.02 ± 0.00^b^	0.01 ± 0.00^de^	0.02 ± 0.00^c^	0.02 ± 0.00^c^
	*Bifidobacterium adolescentis*	1.74 ± 0.08^e^	0.38 ± 0.02 ^g^	4.73 ± 0.04^b^	3.99 ± 0.14^c^	1.13 ± 0.03 ^f^	5.59 ± 0.17^a^	3.61 ± 0.22^d^
	*Sutterella wadsworthensis*	0.46 ± 0.01^d^	0.27 ± 0.01^e^	1.45 ± 0.04^a^	0.98 ± 0.02^b^	0.29 ± 0.03^e^	0.51 ± 0.01^d^	0.80 ± 0.06^c^
	*Lachnospiraceae bacteriu*m 8_1_57FAA	0.54 ± 0.16^a^	0.00 ± 0.01^b^	0.02 ± 0.01^b^	0.01 ± 0.02^b^	ND	ND	0.00 ± 0.01^b^
Health-scarce	*Eggerthella lenta*	0.03 ± 0.00^ａ^	0.02 ± 0.00^ｂ^	0.01 ± 0.00^ｃ^	0.00 ± 0.00^ｄ^	ND	ND	0.00 ± 0.00^ｄ^
	*Ruminococcus gnavus*	1.66 ± 0.04^ａ^	0.08 ± 0.01^ｄ^	0.05 ± 0.01^ｄｅ^	0.32 ± 0.01^ｃ^	0.02 ± 0.00^ｅ^	0.07 ± 0.01^ｄ^	1.50 ± 0.03^ｂ^
	*Anaerotruncus colihominis*	0.03 ± 0.00^ａ^	0.00 ± 0.00^ｂ^	ND	ND	ND	ND	ND
	*Clostridium ramosum*	ND	0.00 ± 0.00^ｄ^	0.00 ± 0.00^ｄ^	0.05 ± 0.00^ｂ^	0.00 ± 0.00^ｄ^	0.03 ± 0.00^ｃ^	0.22 ± 0.02^ａ^
	*Clostridium clostridioforme*	0.00 ± 0.00^ｂ^	0.04 ± 0.01^ａ^	ND	ND	ND	0.00 ± 0.00^ｂ^	ND
	*Streptococcus australis*	0.02 ± 0.00^ａ^	0.01 ± 0.00^ｂ^	ND	ND	ND	ND	0.00 ± 0.00^ｂ^
	*Lachnospiraceae bacteriu*m 5_1_57FAA	ND	0.00 ± 0.00^ｄ^	0.01 ± 0.00^ｂ^	0.01 ± 0.00^ｃ^	0.00 ± 0.00^ｄ^	ND	0.01 ± 0.00^ａ^
	*Clostridium asparagiforme*	0.02 ± 0.00^ｃ^	0.03 ± 0.00^ａ^	0.00 ± 0.00^ｄ^	0.03 ± 0.00^ａ^	ND	0.02 ± 0.00^ｂ^	ND
	*Lachnospiraceae bacterium* 2_1_58FAA	0.04 ± 0.01^ａ^	ND	ND	0.00 ± 0.00^ｃ^	ND	ND	0.01 ± 0.00^ｂ^
	*Fusobacterium nucleatum*	0.00 ± 0.00^ａ^	ND	ND	ND	ND	ND	ND
	*Streptococcus sanguinis*	0.00 ± 0.00^ａ^	ND	ND	ND	ND	ND	ND
	*Blautia producta*	ND	ND	0.00 ± 0.00^ａ^	ND	ND	0.00 ± 0.00^ｂ^	ND
	*Clostridium nexile*	0.07 ± 0.01^ｃ^	ND	0.05 ± 0.01^ｄ^	0.01 ± 0.00^ｅ^	0.06 ± 0.00^ｄ^	0.12 ± 0.01^ｂ^	0.14 ± 0.01^ａ^
	*Klebsiella pneumoniae*	0.03 ± 0.01^ｄ^	0.26 ± 0.05^ｃ^	0.16 ± 0.03^ｃ^	0.19 ± 0.04^ｃ^	0.58 ± 0.13^ｂ^	0.90 ± 0.11^ａ^	0.51 ± 0.02^ｂ^
	*Ruminococcaceae bacteriu*m D16	0.11 ± 0.01^ａ^	0.01 ± 0.00^ｂ^	ND	ND	ND	ND	ND
	*Clostridium symbiosum*	0.10 ± 0.01^ｅ^	2.57 ± 0.01^ａ^	2.17 ± 0.05^ｂ^	0.80 ± 0.01^ｃ^	0.83 ± 0.01^ｃ^	0.59 ± 0.02^ｄ^	0.84 ± 0.02^ｃ^
	*Erysipelotrichaceae bacteri*um 2_2_44A	0.00 ± 0.00^ａ^	ND	ND	ND	ND	ND	ND
	*Lachnospiraceae bacteriu*m 1_4_56FAA	0.01 ± 0.00^ａ^	ND	ND	ND	0.01 ± 0.00^ｂ^	ND	0.00 ± 0.00^ｃ^
	*Clostridium bolteae*	0.09 ± 0.01^ｂ^	0.12 ± 0.01^ａ^	0.01 ± 0.00^ｃ^	0.00 ± 0.00^ｄ^	0.02 ± 0.00^ｃ^	0.00 ± 0.00^ｄ^	0.00 ± 0.00^ｄ^
	*Lachnospiraceae bacteriu*m 3_1_57FAA_CT1	ND	ND	ND	ND	ND	0.17 ± 0.01^ａ^	0.01 ± 0.00^ｂ^
	*Clostridiales bacterium* 1_7_47FAA	ND	0.00 ± 0.00^ａ^	0.00 ± 0.00^ｂ^	0.00 ± 0.00^ｃ^	0.00 ± 0.00^ｃ^	0.00 ± 0.00^ｂ^	0.00 ± 0.00^ｂ^
	*Veillonella atypica*	0.02 ± 0.00^ｄ^	ND	0.21 ± 0.01^ｃ^	0.57 ± 0.01^ｂ^	ND	0.88 ± 0.00^ａ^	ND
	*Clostridium hathewayi*	0.03 ± 0.00^ｆ^	0.66 ± 0.01^ａ^	0.01 ± 0.01^ｄ^	0.02 ± 0.00^ｆ^	0.21 ± 0.00^ｃ^	0.07 ± 0.02^ｅ^	0.46 ± 0.02^ｂ^
	*Streptococcus infantis*	0.00 ± 0.00^ａ^	ND	ND	ND	ND	ND	ND
	*Lachnospiraceae bacteriu*m 9_1_43BFAA	0.00 ± 0.00^ｂ^	ND	ND	ND	ND	ND	0.00 ± 0.00^ａ^
	*Holdemania filiformis*	0.04 ± 0.00^ｃ^	0.08 ± 0.01^ｂ^	0.09 ± 0.01^ａ^	0.02 ± 0.00^ｄ^	0.01 ± 0.00^ｅ^	0.02 ± 0.00^ｄ^	0.03 ± 0.00^ｃ^
	*Clostridium citroniae*	0.02 ± 0.01^ｄ^	0.49 ± 0.02^ａ^	0.19 ± 0.00^ｂ^	0.04 ± 0.01^ｃ^	0.00 ± 0.00^ｅ^	0.02 ± 0.00^ｄｅ^	ND
	*Subdoligranulum* sp. 4_3_54A2FAA	0.01 ± 0.01^e^	0.07 ± 0.00^ｄ^	0.08 ± .0.01^b^	0.07 ± 0.00^bc^	0.07 ± 0.01^c^	0.04 ± 0.00^d^	0.12 ± 0.01^ａ^

*NS0* no substrate addition at 0 h, *NS24* no substrate addition for 24 h, *FS24* fructooligosaccharide fermentation for 24 h, *IN24* inulin fermentation for 24 h, *GS24* galactooligosaccharide fermentation for 24 h, *XS24* xylooligosaccharide fermentation for 24 h, *FL24* 2’-fucosyllactose fermentation for 24 h. Relative abundances are presented in % mean ± SD, and all values are rounded to the second decimal place.

Relative abundances are presented in % mean ± SD, and all values are rounded to the second decimal place. ND, not detected in the respective study group. Different letters next to relative abundance values indicate significant differences among the study groups by Tukey’s honestly significant difference (HSD) mean comparison test (*P* <0.05).

The principal component analysis (PCA) plot in Fig. 1c revealed differences among the NS0 and NS24 (i.e., 24 h incubation with no substrate) control groups and the five prebiotics-treated groups (FS24, IN24, GS24, XS24, and FL24). Notably, the groups treated with prebiotics clustered relatively close to each other, but were clearly distinguishable from the NS0 and NS24 groups; this may signify that the five different types of prebiotics could have had generally similar effects on the overall in vitro growth of the fecal microbial community, although substrate-specific differences were apparent. In summary, these results confirm that, at least in in vitro fermentation settings, prebiotic intake indeed stimulates the growth of gut microbial species frequently observed in healthy conditions.

### GMWI after in vitro fermentation of oligosaccharides

**Fig. 2:**
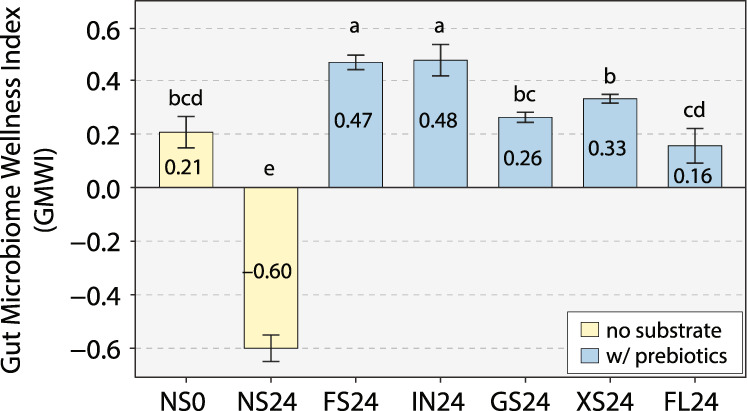
Gut Microbiome Wellness Index (GMWI) of microbial communities after in vitro fecal fermentation of prebiotics for 24 h. Height of bars represent mean GMWI with error bars representing standard deviation from the mean (*n* = 3). Bars with different small letter(s) denote groups with significant differences in GMWI by Tukey’s HSD test (*P* < 0.05). Five prebiotics groups: FS24 fructooligosaccharide fermentation for 24 h, IN24 inulin fermentation for 24 h, GS24 galactooligosaccharide fermentation for 24 h, XS24 xylooligosaccharide fermentation for 24 h, FL24 2’-fucosyllactose fermentation for 24 h. Two control groups: NS0 no substrate addition at 0 h, NS24 no substrate addition for 24 h.

Relative abundances of the 33 Health-prevalent and Health-scarce species detected in the seven study groups (NS0, NS24, FS24, IN24, GS24, XS24, and FL24) are summarized in Table 1. Based on these data, we calculated GMWI for each of the 21 fermented fecal samples (“Methods“). As shown in Fig. 2, the five prebiotics tested in this study resulted in positive average GMWI values—suggesting a healthy, disease-free state—after 24 h fermentation: IN (0.48 ± 0.06), FOS (0.47 ± 0.03), XOS (0.33 ± 0.02), GOS (0.26 ± 0.02), and 2FL (0.16 ± 0.06). Hence, these prebiotics exerted a prebiotic effect on the microbiomes by increasing the collective abundance of Health-prevalent species over that of Health-scarce species (see “Methods” for interpreting the sign of GMWI values). On the other hand, the samples with no substrate addition at 0 h (NS0) and for 24-h incubation were found to have an average GMWI of 0.21 ± 0.06. and –0.60 ± 0.05, respectively (a negative GMWI suggests a non-healthy, disease-harboring state). Only FOS and IN resulted in significantly higher GMWI compared to NS0, yet all five prebiotics led to significantly higher GMWI in relation to NS24 (*P* < 0.05, Tukey’s HSD test).

**Fig. 3:**
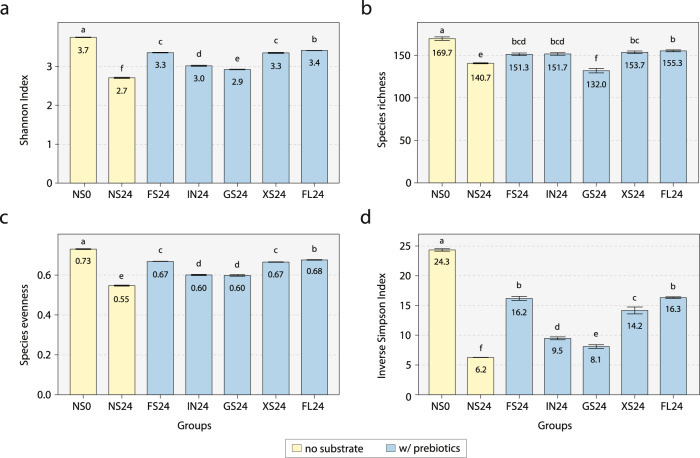
α-diversities of microbial communities after in vitro fecal fermentation of prebiotics for 24 h. Height of bars represent mean (**a**) Shannon Index, (**b**) species richness, (**c**) species evenness, and (**d**) Inverse Simpson Index with error bars representing standard deviation from the mean (*n* = 3). Bars with different small letter(s) denote groups with significant differences in α-diversity by Tukey’s HSD test (*P* < 0.05). Five prebiotics groups: FS24, fructooligosaccharide fermentation for 24 h; IN24 inulin fermentation for 24 h, GS24 galactooligosaccharide fermentation for 24 h, XS24 xylooligosaccharide fermentation for 24 h, FL24 2’-fucosyllactose fermentation for 24 h. Two control groups: NS0 no substrate addition at 0 h, NS24 no substrate addition for 24 h.

Next, α-diversity metrics, which are commonly used as proxies for gut microbiome health, were measured following prebiotic fermentation (Fig. 3a–d). Strikingly, as opposed to the GMWI results, the Shannon Index, species richness, and species evenness, and inverse Simpson’s Index were found to have significantly lower values in all five prebiotic treatment groups compared to the NS0 group (*P* < 0.05, Tukey’s HSD test). Nevertheless, as seen in the GMWI results, these α-diversities in the prebiotics-treated groups were still found to be higher than those of the NS24 group.

Table 2 compares Palframan et al.’s PI (using values found by others^13,24–29^) and GMWI after in vitro fecal fermentation of oligosaccharides for 24 h. Apart from inconsistences observed for NS24, the signs of the original PI and GMWI (which dictate whether there was a prebiotic effect or not) are by and large consistent. In addition, the values of both indices are higher in the prebiotics-treated groups than in the NS24 group. These results provide further impetus for using GMWI as an alternative indexing system for evaluating the prebiotic effect of food components. Moreover, as a reflection of recent advancements in gut microbiome research, GMWI could offer a more state-of-the-art predictor compared to traditional indices.

**Table 2:** Comparative analysis of Palframan et al.’s Prebiotic Index and Gut Microbiome Wellness Index (GMWI) after in vitro fecal fermentation of oligosaccharides for 24 h.

Index	NS24	FS24	IN24	XS24	GS24	2FL	Reference
GMWI	−0.60 ± 0.05	0.47 ± 0.03	0.48 ± 0.06	0.33 ± 0.02	0.26 ± 0.02	0.16 ± 0.06	This study
Prebiotic Index	–	0.77	−1.20	–	3.38	–	Palframan et al.^13^
	–	2.31	1.82	2.19	3.76	–	Rycroft et al.^24^
	1.02	7.64	–	–	–	–	Sanz et al.^25^
	–	2.38	0.88	–	–	–	Ghoddusi et al.^26^
	0.95	3.49	–	–	–	–	Mandalari et al.^27^
	0.10	2.35	–	–	–	–	Moon et al.^28^
	−0.52 ± 0.32	7.93 ± 0.73	–	–	–	–	Massa et al.^29^

*NS24* no substrate addition for 24 h, *FS24* fructooligosaccharide fermentation for 24 h, *IN24* inulin fermentation for 24 h, *XS24* xylooligosaccharide fermentation for 24 h, *GS24* galactooligosaccharide fermentation for 24 h, *FL24* 2’-fucosyllactose fermentation for 24 h.

Relative abundances are presented in % mean ± SD (when available), and all values are rounded to the second decimal place. ‘−’ indicates no available information. Numbers in superscripts correspond to their designated study in the “References” section.

## Discussion

Palframan et al. first introduced the prebiotic index (PI) by accounting for the number of Bifidobacteria (Bif), Bacteroides (Bac), Lactobacilli (Lac), Clostridia (Clos), and total bacteria (Total) as follows^13^:

1 $${PI}=\frac{{Bif}}{{Total}}-\frac{{Bac}}{{Total}}+\frac{{Lac}}{{Total}}-\frac{{Clos}}{{Total}},$$

where Bifidobacteria and Lactobacilli were assumed to be beneficial to health, and Bacteroides and Clostridia to be harmful. Additionally, a modified PI (PIm)^14^ was proposed using maximum growth rates (*μ*_*max*_) as such:

2 $${PIm}={\mu }_{\max }{Bif}+{\mu }_{\max }{Lac}+{\mu }_{\max }{Eub}+{\mu }_{\max }{Bac}+{\mu }_{\max }{Clos}-{\mu }_{\max }{EC}-{\mu }_{\max }{SRB},$$

where Bifidobacteria, Lactobacilli, Eubacteria (Eub), and Bacteroides were regarded as beneficial, and Clostridia, Escherichia coli (EC), and sulfate-reducing bacteria (SRB) as harmful. However, during the formulation of Eqs. (1) and (2), there were no clear guidelines in the selection of beneficial or harmful gut bacteria. Thus, there has been a strong need for a more reliable indexing system based on data-driven and evidence-based approaches.

Herein, we employed GMWI to evaluate the impact of five representative prebiotic ingredients (fructooligosaccharides (FOS), galactooligosaccharides (GOS), xylooligosaccharides (XOS), inulin (IN), and 2’-fucosyllactose (2FL)) on the human gut microbiome. Similar to IN polymers, FOS are oligosaccharides with β(2 → 1) fructosyl-fructose glycosidic bonds, and are resistant to digestive enzymes^30^. These substrates can promote the growth of potentially beneficial bacteria that can produce SCFAs, such as *Lactobacillus* and *Bifidobacterium* spp.^31,32^. GOS are another type of non-digestible oligosaccharides with β(1 → 6) galactosyl bonds to the reducing terminal of β(1 → 6)-linked glucose, and are produced by enzyme reactions using β-galactosidase and lactose^33^. XOS contains two-to-six molecules of xylose with β(1 → 4) linkages produced by hydrolyzing xylan in hemicellulose^34^. 2FL is a trisaccharide composed of l-fucose, d-galactose, and d-glucose units; and is the most prevalent oligosaccharide naturally present in human breast milk^23^.

Several limitations of our study should be noted when interpreting the results. First, we conducted our metagenomic analyses mainly using species-level abundances, even though the strain level is a more clinically informative taxonomic rank. Nevertheless, the shotgun metagenomic approach used in this study provides enhanced detection of bacterial species compared to 16S rRNA gene amplicon sequencing^35,36^. Second, metagenomic functional profiles were not considered in this study, as genes for biochemical reaction potential are not incorporated into the current version of GMWI. Third, the use of GMWI is limited to adult studies only, since its original formulation excluded samples from subjects who were less than 10 years of age. Possibly related to this reason, the 2FL-treated samples tended to show relatively lower GMWI (0.16 ± 0.06) than the other tested prebiotics. Last, all results in this study were obtained using an in vitro fermentation model system. However, despite their limitations in mimicking in vivo physiology, in vitro models provide an obvious convenience regarding experimentation, and, importantly, overcome some important hurdles that accompany in vivo human studies, such as ethical compliance and high drop-out rates^37^. Furthermore, such in vitro models may be instrumental in validating the statistical (based on inference) and mechanistic (based on prior biological knowledge) modeling techniques of gut microbiome ecosystems previously proposed by our group^38–41^.

The academic discipline of food science and human nutrition is currently being revolutionized by recent progresses in big data analytics and large-scale computation^42–44^. Along these lines, the results presented in this study suggest that GMWI can serve as a novel quantitative tool to guide dietary intervention studies. As the impact of the gut microbiome on disease onset and progression has been widely recognized (and in certain cases even validated)^45–47^, there is now emerging evidence showing that dietary or nutritional modulation of the gut microbiome may have a future role in disease prevention and treatment^48^. Looking ahead, the methodology and results demonstrated herein could have implications for designing personalized diets by closely monitoring the prebiotic effect of food components on gut microbiome wellness.

## Methods

### Raw materials

The commercial prebiotics used in this study were fructooligosaccharides (FOS) (95.38% purity, Samyang Co., Seoul, Korea), galactooligosaccharides (GOS) (≥98.99% purity, Genofocus Co., Daejeon, Korea), xylooligosaccharides (XOS) (≥95.20% purity, Desang Co., Incheon, Korea), inulin (IN) (≥90% purity, Frutafit HD, Sensus, Netherlands), and 2’-fucosyllactose (2FL) (≥94% purity, APTechnology Corp., Suwon, Korea) of food grade. All other chemicals used in this study were of analytical grade. Vitamin K_1_ was purchased from Wako Pure Chemical Industries Ltd. (Osaka, Japan). Water peptone and yeast extract were purchased from BD Biosciences (Franklin Lakes, NJ, USA). CaCl_2_ ∙ 2H_2_O, K_2_HPO_4_, KH_2_PO_4_, and NaCl were purchased from Junsei (Tokyo, Japan). Bile salts, L-cysteine hydrochloride, hemin, MgSO_4_ ∙ 7H_2_O, and NaHCO_3_ were purchased from Sigma-Aldrich (St. Louis, MO, USA). Tween 80 was purchased from VWR (Radnor, PA, USA).

### In vitro fecal fermentation

In vitro fecal fermentation (24 h) of commercial prebiotics (FOS, GOS, XOS, IN, and 2FL) was conducted according to an established protocol^49^. More specifically, 300 mL capacity water-jacketed fermenter vessels and basal growth medium (2 g/L peptone water, 1 g/L yeast extract, 0.1 g/L NaCl, 0.04 g/L K_2_HPO_4_, 0.04 g/L KH_2_PO_4_, 0.01 g/L MgSO_4_·7H_2_O, 0.01 g/L CaCl_2_·2H_2_O, 2 g/L NaHCO_3_, 0.5 g/L bile salts, 0.5 g/L L-cysteine hydrochloride, 50 mg/L hemin, 10 μL/L vitamin K_1_, and 2 mL/L Tween 80) were used for batch fermentation. A total of 135 mL of this medium was inoculated with 15 mL of 10% (w/v) mixed human fecal slurry, prepared by mixing and homogenizing freshly voided adult feces in 0.1 M PBS (pH 7.0). Fecal samples were obtained from 19 healthy adult volunteers (14 males and 5 females, age from 25 to 30 years) who were not consuming antibiotics or pre/probiotics at the time of the study, and had no recent history of gastrointestinal disorders. All participants involved in this study provided written informed consent prior to sample collection. The feces were collected and mixed under anaerobic conditions. The Institutional Review Board of Chungbuk National University approved the study protocol and consent form (CBNU-201905-BR-839-01). FOS, GOS, XOS, IN, and 2FL were added at a final concentration of 1% (w/v). The slurry in each vessel was magnetically stirred, and the pH and temperature were maintained at pH 6.8 and 37 °C, respectively. The anaerobic conditions were maintained by sparging the vessels with oxygen-free nitrogen gas at a flow rate of 15 mL/min. For each of the seven study groups (NS0, NS24, FS24, IN24, GS24, XS24, and FL24), samples (5 mL) were collected in triplicates for metagenomic sequencing analysis.

### Shotgun metagenome sequencing

Metagenomic DNA isolated from fecal samples was purified and subjected to quality-control assessments, including a DNA purity test using a Nanodrop (OD_260_/OD_280_). Subsequently, shotgun metagenomic libraries were constructed using the NEBNext Ultra II DNA Library Prep Kit for Illumina (New England Biolabs, Ipswich, MA, USA) according to the manufacturer’s protocols. Libraries were generated and sequenced in paired-end mode of 150 base pairs (2 × 150 bp) using the Illumina NovaSeq6000 System at Macrogen (Seoul, Korea). Sequence reads were processed with the KneadData v0.5.1 quality-control pipeline (http://huttenhower.sph.harvard.edu/kneaddata), which uses Trimmomatic v0.39 and Bowtie2 v0.1 to remove low-quality read bases and human reads, respectively. Trimmomatic v0.39 was run with parameters SLIDINGWINDOW:4:30, and Phred quality scores were thresholded at “<30.” Illumina adapter sequences were removed, and trimmed nonhuman reads shorter than 36 bp in nucleotide length were discarded. Taxonomic profiling was conducted using the MetaPhlAn2 v2.7.0 taxonomic sequence classification system with default parameters.

### GMWI calculation

The Gut Microbiome Wellness Index (GMWI) was calculated using GMWI-webtool, a user-friendly browser application that computes GMWI from shotgun metagenome taxonomic profiles of stool^50^. Briefly, 50 gut microbial species associated with healthy (i.e., absent of disease) and non-healthy (i.e., having a clinical diagnosis of at least one disease) people were used to formulate GMWI. These 50 microbial species are classified as either 7 Health-prevalent or 43 Health-scarce species based on their prevalence in the gut microbiomes (shotgun metagenomes) of 4347 human subjects.

The GMWI for a given sample is calculated based on the following mathematical formulas: For the set of Health-prevalent species *M*_*H*_, its “collective abundance” ${\psi }_{{M}_{H}}$ is defined as:

3 $${\psi }_{{M}_{H}}=\frac{{R}_{{M}_{H}}}{\left|{M}_{H}\right|}\sum _{j\in {I}_{{M}_{H}}}\left|{n}_{j}{\rm{ln}}\left({n}_{j}\right)\right|,$$

where ${R}_{{M}_{H}}$ is the richness of *M*_*H*_ species in a sample, $\left|{M}_{H}\right|$ is the set size of *M*_*H*_, ${I}_{{M}_{H}}$ is the index set of *M*_*H*_, and *n*_*j*_ is the relative abundance of species $j$. To compute GMWI, the collective abundances of species in sets *M*_*H*_ (Health-prevalent) and *M*_*N*_ (Health-scarce) are compared using a log-ratio of ${\psi }_{{M}_{H}}$ to ${\psi }_{{M}_{N}}$ in:

4 $${h}_{{M}_{H,}{M}_{N}}={{\rm{log }}}_{10}\left(\frac{{\psi }_{{M}_{H}}}{{\psi }_{{M}_{N}}}\right),$$

where a positive value of GMWI suggests that microbes associated with a healthy, disease-free state dominate the sample over microbes associated with a non-healthy, disease-harboring state; and a zero value indicates that there is an equal balance of both species sets. Therefore, the GMWI can be interpreted as the degree to which a given gut microbiome sample portrays a higher collective abundance of the 7 Health-prevalent species over the 43 Health-scarce species.

### Statistical analysis

Relative abundances of the microbial species in each study group are presented as mean ± standard deviation (SD) of the triplicate samples unless indicated otherwise. One-way analysis of variance (ANOVA) was used to determine statistically significant differences between study groups. Tukey’s post hoc honestly significant difference (HSD) test was applied to all pair-wise group comparisons to identify significantly different means (*P* < 0.05).

### Reporting summary

Further information on research design is available in the Nature Research Reporting Summary linked to this article.

## Supplementary information

Supplementary Information

Reporting Summary

## Data Availability

The raw sequence reads obtained for this study are deposited at https://www.ncbi.nlm.nih.gov/sra/PRJNA902022.”. and can be accessed without restrictions. The remaining data from the current study are available in the main text or in the supplementary information.
