# Supplementary material for: Evaluating the prebiotic effect of oligosaccharides on gut microbiome wellness using in vitro fecal fermentation
Source: NPJ Sci Food. 2023 May 9;7:18. doi: 10.1038/s41538-023-00195-1 (PMC10170090; doi:10.1038/s41538-023-00195-1)
Supplement: Supplementary file 1 — Supplementary Information [file 41538_2023_195_MOESM1_ESM.pdf]

Supplementary Information

Supplemental Table 1. Relative abundance after *in vitro* fecal fermentation for 24 h

Species	NS0_1	NS0_2	NS0_3	NS24_1	NS24_2	NS24_3	FS24_1	FS24_2	FS24_3	IN24_1	IN24_2	IN24_3	GS24_1	GS24_2	GS24_3	XS24_1	XS24_2	XS24_3	FL24_1	FL24_2	FL24_3
Actinomyces_odontolyticus	0.01	0.02	0.02	0.00	0.00	0.00	0.00	0.00	0.00	0.00	0.00	0.00	0.00	0.00	0.00	0.00	0.00	0.00	0.00	0.00	0.00
Actinomyces_sp_HPA0247	0.00	0.00	0.00	0.00	0.00	0.00	0.00	0.00	0.00	0.00	0.00	0.00	0.00	0.00	0.00	0.00	0.00	0.00	0.00	0.00	0.00
Actinomyces_sp_ICM39	0.02	0.02	0.02	0.00	0.00	0.00	0.00	0.00	0.00	0.00	0.00	0.00	0.00	0.00	0.00	0.00	0.00	0.00	0.00	0.00	0.00
Actinomyces_sp_ICM47	0.01	0.01	0.02	0.00	0.00	0.00	0.00	0.00	0.00	0.00	0.00	0.00	0.00	0.00	0.00	0.00	0.00	0.00	0.00	0.00	0.00
Actinomyces_sp_oral_taxon_172	0.00	0.00	0.00	0.00	0.00	0.00	0.00	0.00	0.00	0.00	0.00	0.00	0.00	0.00	0.00	0.00	0.00	0.00	0.00	0.00	0.00
Rothia_mucilaginosa	0.08	0.07	0.08	0.00	0.00	0.00	0.00	0.00	0.00	0.00	0.00	0.00	0.00	0.00	0.00	0.00	0.00	0.00	0.00	0.00	0.00
Bifidobacterium_adolescentis	1.66	1.76	1.81	0.37	0.39	0.39	4.58	4.86	4.74	3.83	4.12	4.00	1.10	1.16	1.13	5.41	5.76	5.60	3.39	3.83	3.61
Bifidobacterium_animalis	0.00	0.00	0.00	0.00	0.00	0.00	0.00	0.00	0.00	0.00	0.00	0.00	0.00	0.00	0.00	0.00	0.00	0.00	0.00	0.00	0.00
Bifidobacterium_bifidum	1.58	1.84	1.84	0.12	0.12	0.13	1.33	1.36	1.32	0.27	0.29	0.28	0.68	0.76	0.73	0.18	0.20	0.20	0.30	0.35	0.35
Bifidobacterium_catenulatum	0.03	0.02	0.02	0.00	0.00	0.00	0.18	0.19	0.17	0.07	0.08	0.08	0.17	0.17	0.18	0.06	0.06	0.07	0.04	0.05	0.04
Bifidobacterium_longum	1.58	1.70	1.69	0.36	0.38	0.40	6.53	6.83	6.58	1.86	1.97	1.85	4.79	5.02	4.94	2.14	2.29	2.22	0.73	0.86	0.80
Bifidobacterium_pseudocatenulatum	0.09	0.10	0.11	0.04	0.05	0.05	1.33	1.34	1.33	0.34	0.34	0.31	1.35	1.31	1.38	0.60	0.64	0.67	0.83	0.89	0.84
Bifidobacterium_sp_12_1_47BFAA	0.00	0.00	0.02	0.00	0.00	0.01	0.02	0.01	0.03	0.00	0.00	0.00	0.02	0.04	0.06	0.00	0.00	0.00	0.00	0.02	0.00
Adlercreutzia_unclassified	0.00	0.00	0.00	0.00	0.00	0.00	0.00	0.00	0.00	0.00	0.00	0.00	0.00	0.00	0.00	0.00	0.00	0.00	0.00	0.00	0.00
Atopobium_sp_ICM58	0.00	0.01	0.01	0.00	0.00	0.00	0.00	0.00	0.00	0.00	0.00	0.00	0.00	0.00	0.00	0.00	0.00	0.00	0.00	0.00	0.00
Collinsella_aerofaciens	0.46	0.52	0.50	0.15	0.16	0.16	0.28	0.31	0.28	1.27	1.40	1.33	0.05	0.05	0.04	0.14	0.14	0.14	5.35	6.03	5.88
Eggerthella_lenta	0.04	0.03	0.03	0.01	0.02	0.02	0.01	0.01	0.01	0.00	0.00	0.00	0.00	0.00	0.00	0.00	0.00	0.00	0.00	0.00	0.00
Eggerthella_sp_1_3_56FAA	0.04	0.04	0.03	0.00	0.00	0.00	0.00	0.00	0.01	0.00	0.00	0.00	0.00	0.00	0.00	0.00	0.00	0.00	0.00	0.00	0.00
Eggerthella_sp_HGA1	0.04	0.03	0.02	0.01	0.01	0.00	0.00	0.00	0.00	0.00	0.00	0.00	0.00	0.00	0.00	0.00	0.00	0.00	0.00	0.00	0.00
Eggerthella_unclassified	0.05	0.07	0.09	0.02	0.01	0.03	0.02	0.01	0.00	0.01	0.01	0.01	0.00	0.00	0.00	0.01	0.00	0.00	0.01	0.01	0.01
Bacteroides_caccae	0.10	0.09	0.10	0.02	0.02	0.02	0.05	0.06	0.05	0.24	0.23	0.23	0.04	0.04	0.04	0.04	0.04	0.05	0.04	0.04	0.04
Bacteroides_cellulosilyticus	0.00	0.00	0.00	0.13	0.14	0.14	0.22	0.22	0.21	0.12	0.12	0.12	0.27	0.28	0.28	1.16	1.11	1.11	0.17	0.17	0.17
Bacteroides_coprocola	0.26	0.25	0.26	0.00	0.00	0.00	0.01	0.01	0.01	0.02	0.02	0.03	0.00	0.00	0.00	0.22	0.21	0.21	0.02	0.01	0.01
Bacteroides_coprophilus	0.38	0.39	0.39	0.00	0.00	0.00	0.00	0.00	0.00	0.00	0.00	0.00	0.00	0.00	0.00	0.00	0.00	0.00	0.00	0.00	0.00
Bacteroides_dorei	2.59	2.52	2.53	0.72	0.71	0.74	0.20	0.21	0.19	0.28	0.29	0.30	0.34	0.30	0.30	0.69	0.69	0.66	0.60	0.55	0.55
Bacteroides_eggerthii	0.21	0.20	0.19	0.39	0.35	0.37	0.11	0.12	0.11	0.09	0.09	0.09	0.07	0.07	0.06	0.23	0.24	0.25	0.23	0.22	0.22
Bacteroides_faecis	0.01	0.01	0.01	0.04	0.03	0.04	0.11	0.11	0.10	0.85	0.80	0.84	0.24	0.23	0.22	0.06	0.06	0.07	0.19	0.20	0.20
Bacteroides_finegoldii	0.02	0.02	0.01	0.00	0.00	0.00	0.01	0.01	0.01	0.00	0.00	0.01	0.01	0.01	0.01	0.06	0.06	0.06	0.11	0.10	0.10
Bacteroides_fragilis	1.89	1.82	1.85	2.00	1.89	1.94	2.84	2.74	2.76	1.67	1.62	1.58	3.20	3.02	3.10	0.90	0.89	0.89	4.89	4.77	4.71
Bacteroides_intestinalis	0.00	0.00	0.00	0.25	0.25	0.25	0.36	0.36	0.34	0.37	0.34	0.35	0.21	0.20	0.19	1.65	1.62	1.63	0.67	0.66	0.67
Bacteroides_nordii	0.00	0.00	0.00	0.04	0.04	0.03	0.14	0.14	0.16	0.23	0.25	0.23	0.08	0.08	0.08	0.08	0.07	0.08	0.09	0.10	0.08
Bacteroides_ovatus	0.85	0.78	0.78	1.12	1.07	1.03	1.63	1.53	1.58	1.09	1.09	1.11	1.74	1.67	1.67	4.76	4.67	4.69	2.67	2.56	2.59
Bacteroides_plebeius	0.50	0.47	0.47	0.00	0.00	0.00	0.00	0.00	0.01	0.18	0.19	0.19	0.06	0.06	0.06	0.05	0.05	0.05	0.01	0.01	0.01
Bacteroides_salyersiae	0.00	0.00	0.00	0.12	0.13	0.12	0.84	0.78	0.78	0.47	0.46	0.45	0.24	0.23	0.23	0.39	0.33	0.32	0.79	0.72	0.77
Bacteroides_sp_1_1_14	0.02	0.02	0.02	0.08	0.07	0.08	0.14	0.14	0.14	0.08	0.07	0.08	0.12	0.12	0.10	0.12	0.11	0.12	0.14	0.12	0.13
Bacteroides_sp_1_1_30	0.09	0.09	0.10	0.09	0.09	0.07	0.15	0.15	0.15	0.15	0.13	0.13	0.08	0.09	0.08	0.34	0.32	0.31	0.18	0.13	0.14
Bacteroides_sp_1_1_6	0.04	0.04	0.03	0.18	0.16	0.17	0.30	0.34	0.31	0.35	0.32	0.32	0.33	0.30	0.32	0.27	0.25	0.27	0.33	0.34	0.39
Bacteroides_sp_2_1_16	0.01	0.01	0.01	0.01	0.01	0.02	0.02	0.02	0.02	0.01	0.01	0.01	0.04	0.04	0.04	0.01	0.01	0.01	0.03	0.02	0.03
Bacteroides_sp_2_1_22	0.00	0.08	0.00	0.34	0.20	0.32	0.53	0.45	0.49	0.15	0.16	0.13	0.15	0.13	0.13	0.10	0.13	0.09	0.42	0.30	0.24
Bacteroides_sp_2_1_33B	0.01	0.01	0.01	0.01	0.01	0.01	0.01	0.01	0.01	0.01	0.01	0.01	0.02	0.01	0.02	0.01	0.01	0.01	0.01	0.01	0.01
Bacteroides_sp_2_1_56FAA	0.20	0.19	0.16	0.23	0.22	0.24	0.34	0.31	0.32	0.22	0.19	0.21	0.68	0.65	0.65	0.11	0.11	0.11	0.61	0.58	0.59
Bacteroides_sp_2_2_4	0.01	0.01	0.00	0.01	0.01	0.01	0.02	0.02	0.02	0.05	0.02	0.02	0.02	0.02	0.02	0.05	0.05	0.05	0.03	0.03	0.03
Bacteroides_sp_3_1_19	0.20	0.19	0.20	0.15	0.15	0.18	0.09	0.09	0.10	0.13	0.10	0.10	0.24	0.25	0.24	0.18	0.21	0.21	0.15	0.14	0.15
Bacteroides_sp_3_1_23	0.03	0.03	0.03	0.05	0.04	0.05	0.11	0.10	0.10	0.08	0.09	0.08	0.08	0.07	0.08	0.18	0.17	0.19	0.12	0.11	0.12
Bacteroides_sp_3_1_33FAA	0.07	0.07	0.06	0.01	0.00	0.00	0.00	0.00	0.00	0.01	0.01	0.00	0.01	0.00	0.00	0.01	0.01	0.01	0.01	0.01	0.00
Bacteroides_sp_3_1_40A	0.48	0.49	0.50	0.18	0.15	0.17	0.14	0.15	0.12	0.11	0.13	0.13	0.15	0.14	0.14	0.28	0.33	0.29	0.25	0.23	0.24
Bacteroides_sp_3_2_5	0.00	0.00	0.00	0.01	0.01	0.00	0.01	0.01	0.02	0.00	0.00	0.00	0.05	0.04	0.05	0.00	0.00	0.00	0.04	0.03	0.03
Bacteroides_sp_4_1_36	0.00	0.01	0.01	0.02	0.02	0.03	0.02	0.02	0.02	0.03	0.03	0.03	0.01	0.01	0.01	0.02	0.02	0.02	0.03	0.03	0.03
Bacteroides_sp_4_3_47FAA	0.13	0.11	0.11	0.05	0.04	0.06	0.02	0.02	0.02	0.03	0.02	0.02	0.01	0.02	0.02	0.08	0.06	0.07	0.06	0.05	0.06
Bacteroides_sp_9_1_42FAA	0.04	0.05	0.06	0.04	0.04	0.03	0.01	0.01	0.01	0.01	0.01	0.02	0.02	0.01	0.02	0.02	0.02	0.01	0.01	0.02	0.01
Bacteroides_sp_D2	0.00	0.00	0.00	0.00	0.00	0.00	0.00	0.00	0.00	0.00	0.00	0.00	0.00	0.00	0.00	0.01	0.01	0.00	0.00	0.00	0.00
Bacteroides_sp_D20	0.07	0.07	0.08	0.24	0.24	0.25	0.27	0.26	0.23	0.42	0.42	0.40	0.05	0.05	0.05	0.30	0.33	0.32	0.46	0.42	0.40
Bacteroides_sp_D22	0.01	0.01	0.01	0.01	0.01	0.02	0.00	0.00	0.00	0.00	0.00	0.00	0.00	0.00	0.00	0.00	0.00	0.00	0.00	0.00	0.00
Bacteroides_sp_HPS0048	0.00	0.00	0.00	0.00	0.00	0.00	0.00	0.00	0.00	0.00	0.00	0.00	0.00	0.00	0.00	0.00	0.00	0.00	0.00	0.00	0.00
Bacteroides_stercoris	0.09	0.08	0.08	0.04	0.04	0.04	1.19	1.19	1.16	0.37	0.36	0.37	0.76	0.72	0.71	0.74	0.72	0.69	0.31	0.30	0.31
Bacteroides_thetaiotaomicron	0.63	0.60	0.63	5.06	4.88	4.89	12.46	11.93	12.03	21.64	20.72	20.82	9.75	9.14	9.15	6.07	6.03	5.97	11.69	11.19	11.39
Bacteroides_uniformis	2.05	2.13	2.06	8.12	7.95	7.69	7.41	7.45	7.72	14.75	13.80	14.41	4.90	4.81	4.75	9.99	9.53	9.54	11.01	10.41	10.53
Bacteroides_vulgatus	10.52	10.17	9.84	1.35	1.32	1.34	1.05	1.09	1.07	1.24	1.18	1.20	3.16	2.96	3.03	2.92	2.83	2.80	1.96	1.89	1.85
Bacteroides_xylanisolvens	0.23	0.18	0.20	0.21	0.20	0.20	0.22	0.20	0.20	0.41	0.36	0.36	0.12	0.10	0.11	0.79	0.73	0.70	0.42	0.42	0.44
Bacteroidales_noname_unclassified	0.19	0.21	0.21	0.01	0.02	0.02	0.07	0.07	0.07	0.04	0.05	0.04	0.01	0.02	0.01	0.03	0.03	0.03	0.04	0.04	0.04
Barnesiella_unclassified	0.04	0.04	0.05	0.00	0.00	0.00	0.06	0.06	0.06	0.05	0.05	0.04	0.00	0.00	0.00	0.07	0.06	0.06	0.08	0.08	0.08
Odoribacter_splanchnicus	0.04	0.04	0.05	0.01	0.00	0.01	0.00	0.00	0.00	0.00	0.00	0.00	0.00	0.00	0.00	0.01	0.01	0.01	0.01	0.01	0.01
Odoribacter_unclassified	0.00	0.00	0.00	0.00	0.00	0.00	0.00	0.00	0.00	0.01	0.00	0.00	0.00	0.00	0.00	0.00	0.00	0.00	0.00	0.00	0.00
Parabacteroides_distasonis	1.87	1.92	1.86	1.59	1.55	1.55	1.01	1.02	1.02	1.35	1.35	1.36	2.45	2.44	2.42	2.07	2.08	2.08	1.50	1.46	1.46
Parabacteroides_goldsteinii	0.00	0.00	0.00	0.00	0.00	0.00	0.00	0.00	0.00	0.00	0.00	0.00	0.00	0.00	0.00	0.00	0.00	0.00	0.00	0.01	0.00
Parabacteroides_johnsonii	0.20	0.21	0.21	0.06	0.05	0.05	0.04	0.04	0.04	0.06	0.06	0.06	1.96	1.81	1.81	0.24	0.21	0.22	1.40	1.35	1.36

Parabacteroides_merdae	1.18	1.17	1.19	0.86	0.84	0.86	0.66	0.66	0.67	0.89	0.84	0.85	3.32	3.12	3.18	1.26	1.21	1.23	3.11	3.07	3.07
Parabacteroides_sp_20_3	0.23	0.24	0.20	0.13	0.13	0.12	0.10	0.12	0.11	0.15	0.15	0.13	0.44	0.46	0.44	0.25	0.23	0.25	0.34	0.31	0.32
Parabacteroides_sp_D13	0.07	0.09	0.10	0.13	0.10	0.10	0.05	0.07	0.06	0.05	0.07	0.07	0.10	0.09	0.11	0.08	0.08	0.08	0.07	0.07	0.07
Parabacteroides_sp_D25	0.01	0.01	0.01	0.00	0.01	0.01	0.00	0.00	0.00	0.00	0.00	0.01	0.01	0.01	0.01	0.01	0.01	0.01	0.00	0.00	0.00
Paraprevotella_clara	0.02	0.02	0.01	0.00	0.00	0.00	0.00	0.00	0.00	0.00	0.00	0.00	0.00	0.00	0.00	0.00	0.00	0.00	0.00	0.00	0.00
Paraprevotella_unclassified	0.15	0.15	0.17	0.00	0.00	0.00	0.00	0.00	0.00	0.00	0.00	0.00	0.00	0.00	0.00	0.02	0.02	0.02	0.01	0.01	0.01
Prevotella_copri	0.80	0.70	0.69	0.00	0.00	0.00	0.00	0.00	0.00	0.00	0.00	0.00	0.00	0.00	0.00	0.01	0.01	0.01	0.00	0.00	0.00
Alistipes_finegoldii	0.08	0.07	0.07	0.18	0.15	0.16	0.66	0.74	0.65	0.41	0.44	0.42	0.16	0.15	0.16	0.33	0.34	0.35	0.33	0.32	0.35
Alistipes_indistinctus	0.01	0.02	0.01	0.06	0.06	0.07	0.10	0.10	0.10	0.26	0.27	0.26	0.03	0.03	0.03	0.13	0.14	0.13	0.18	0.20	0.19
Alistipes_onderdonkii	0.17	0.19	0.20	0.56	0.57	0.57	0.94	1.05	0.92	1.15	1.33	1.26	0.29	0.31	0.28	0.60	0.71	0.70	0.90	1.05	1.05
Alistipes_putredinis	1.97	2.03	2.01	0.73	0.73	0.71	0.64	0.64	0.67	0.16	0.15	0.15	0.09	0.10	0.09	0.27	0.28	0.26	0.47	0.47	0.47
Alistipes_senegalensis	0.00	0.01	0.01	0.01	0.01	0.01	0.04	0.04	0.03	0.03	0.02	0.03	0.01	0.01	0.01	0.02	0.02	0.02	0.02	0.02	0.02
Alistipes_shahii	0.07	0.06	0.06	0.14	0.12	0.12	0.87	0.91	0.95	0.23	0.24	0.25	0.16	0.16	0.14	0.26	0.27	0.24	0.29	0.32	0.33
Alistipes_sp_HGB5	0.00	0.00	0.00	0.00	0.00	0.00	0.05	0.05	0.05	0.03	0.02	0.03	0.01	0.01	0.01	0.02	0.02	0.03	0.02	0.02	0.02
Bacillus_amyloliquefaciens	0.02	0.02	0.02	0.00	0.00	0.00	0.00	0.00	0.00	0.00	0.00	0.00	0.00	0.00	0.00	0.00	0.00	0.00	0.00	0.00	0.00
Bacillus_cereus_thuringiensis	0.00	0.00	0.00	0.00	0.00	0.00	0.38	0.29	0.34	0.12	0.14	0.12	0.00	0.00	0.00	0.02	0.02	0.02	0.00	0.00	0.00
Staphylococcus_aureus	0.00	0.00	0.00	0.00	0.00	0.08	0.00	0.00	0.00	0.00	0.00	0.00	0.00	0.00	0.00	0.00	0.00	0.00	0.00	0.00	0.00
Enterococcus_avium	0.00	0.00	0.00	0.00	0.00	0.00	0.00	0.00	0.00	0.00	0.00	0.00	0.00	0.00	0.00	0.00	0.00	0.00	0.00	0.00	0.00
Enterococcus_casseliflavus	0.00	0.00	0.00	0.00	0.00	0.00	0.00	0.00	0.00	0.04	0.03	0.02	0.04	0.02	0.03	0.02	0.01	0.01	0.00	0.00	0.00
Enterococcus_faecalis	0.00	0.00	0.00	0.01	0.01	0.01	0.00	0.00	0.00	0.00	0.00	0.00	0.00	0.00	0.00	0.00	0.00	0.00	0.15	0.11	0.12
Enterococcus_faecium	0.10	0.10	0.10	0.15	0.16	0.22	4.49	3.90	4.63	0.61	0.47	0.68	1.31	1.17	1.29	0.83	0.62	0.69	0.50	0.36	0.46
Enterococcus_hirae	0.00	0.00	0.00	0.00	0.00	0.00	0.00	0.00	0.00	0.00	0.00	0.00	0.00	0.00	0.00	0.00	0.00	0.00	0.00	0.00	0.00
Lactobacillus_acidophilus	0.08	0.08	0.08	0.00	0.01	0.01	0.02	0.01	0.02	0.00	0.02	0.01	0.00	0.00	0.00	0.00	0.00	0.00	0.01	0.01	0.00
Lactobacillus_brevis	0.00	0.00	0.00	0.00	0.00	0.00	0.00	0.00	0.00	0.00	0.00	0.00	0.00	0.00	0.00	0.00	0.00	0.00	0.00	0.00	0.00
Lactobacillus_fructivorans	0.02	0.01	0.02	0.00	0.00	0.00	0.00	0.00	0.00	0.00	0.00	0.00	0.00	0.00	0.00	0.00	0.00	0.00	0.00	0.00	0.00
Lactobacillus_helveticus	0.02	0.02	0.03	0.00	0.00	0.00	0.00	0.00	0.00	0.00	0.00	0.00	0.00	0.00	0.00	0.00	0.00	0.00	0.00	0.00	0.00
Lactobacillus_rhamnosus	0.02	0.02	0.01	0.00	0.00	0.00	0.00	0.00	0.00	0.00	0.00	0.00	0.00	0.00	0.00	0.00	0.00	0.00	0.00	0.00	0.00
Lactobacillus_ruminis	1.49	1.50	1.52	0.04	0.04	0.03	0.73	0.71	0.66	0.11	0.09	0.10	0.07	0.05	0.06	0.43	0.38	0.39	0.03	0.03	0.03
Leuconostoc_lactis	0.08	0.07	0.09	0.01	0.01	0.01	0.04	0.04	0.04	0.01	0.01	0.01	0.02	0.02	0.02	0.07	0.08	0.07	0.00	0.00	0.00
Lactococcus_lactis	0.00	0.00	0.00	0.00	0.00	0.00	0.00	0.00	0.00	0.00	0.00	0.00	0.00	0.00	0.00	0.00	0.00	0.00	0.04	0.05	0.05
Streptococcus_australis	0.02	0.02	0.01	0.01	0.00	0.01	0.00	0.00	0.00	0.00	0.00	0.00	0.00	0.00	0.00	0.00	0.00	0.00	0.00	0.00	0.01
Streptococcus_constellatus	0.00	0.00	0.00	0.00	0.00	0.01	0.00	0.00	0.00	0.00	0.00	0.00	0.00	0.00	0.00	0.00	0.00	0.00	0.00	0.00	0.00
Streptococcus_infantis	0.00	0.00	0.00	0.00	0.00	0.00	0.00	0.00	0.00	0.00	0.00	0.00	0.00	0.00	0.00	0.00	0.00	0.00	0.00	0.00	0.00
Streptococcus_parasanguinis	0.12	0.12	0.11	0.02	0.02	0.02	0.01	0.01	0.00	0.02	0.02	0.02	0.00	0.00	0.00	0.00	0.00	0.00	0.01	0.01	0.01
Streptococcus_salivarius	0.21	0.20	0.20	0.05	0.03	0.04	0.08	0.06	0.06	0.05	0.06	0.05	0.01	0.01	0.01	0.00	0.00	0.00	0.01	0.01	0.01
Streptococcus_sanguinis	0.01	0.00	0.00	0.00	0.00	0.00	0.00	0.00	0.00	0.00	0.00	0.00	0.00	0.00	0.00	0.00	0.00	0.00	0.00	0.00	0.00
Streptococcus_sp_GMD4S	0.00	0.00	0.00	0.00	0.00	0.00	0.00	0.00	0.00	0.00	0.00	0.00	0.00	0.00	0.00	0.00	0.00	0.00	1.36	1.11	1.28
Streptococcus_thermophilus	2.60	2.42	2.62	0.54	0.49	0.53	0.08	0.08	0.06	0.06	0.06	0.06	0.03	0.02	0.03	0.05	0.05	0.05	0.06	0.07	0.07
Clostridiaceae_noname_unclassified	0.00	0.00	0.00	0.02	0.02	0.02	0.00	0.00	0.00	0.01	0.01	0.01	0.06	0.05	0.05	0.02	0.01	0.01	0.00	0.00	0.00
Clostridium_asparagiforme	0.02	0.02	0.02	0.03	0.03	0.04	0.00	0.00	0.00	0.03	0.03	0.03	0.00	0.00	0.00	0.02	0.03	0.03	0.00	0.00	0.00
Clostridium_bolteae	0.10	0.08	0.10	0.12	0.12	0.13	0.01	0.01	0.01	0.00	0.00	0.00	0.01	0.02	0.02	0.00	0.00	0.00	0.00	0.00	0.00
Clostridium_butyricum	0.00	0.00	0.00	0.00	0.00	0.00	0.00	0.00	0.00	0.05	0.05	0.04	0.22	0.21	0.22	0.08	0.08	0.08	0.02	0.02	0.03
Clostridium_citroniae	0.03	0.02	0.01	0.50	0.50	0.47	0.19	0.19	0.19	0.04	0.04	0.03	0.00	0.01	0.00	0.01	0.02	0.02	0.00	0.00	0.00
Clostridium_clostridioforme	0.00	0.00	0.00	0.04	0.03	0.04	0.00	0.00	0.00	0.00	0.00	0.00	0.00	0.00	0.00	0.00	0.00	0.00	0.00	0.00	0.00
Clostridium_hathewayi	0.03	0.03	0.03	0.67	0.64	0.66	0.09	0.10	0.09	0.02	0.02	0.02	0.21	0.20	0.21	0.09	0.08	0.05	0.44	0.47	0.47
Clostridium_leptum	0.74	0.75	0.74	0.05	0.05	0.04	0.01	0.02	0.02	0.00	0.01	0.00	0.00	0.01	0.00	0.40	0.34	0.37	0.00	0.01	0.00
Clostridium_nexile	0.06	0.08	0.07	0.00	0.00	0.00	0.05	0.05	0.04	0.00	0.01	0.00	0.06	0.06	0.05	0.13	0.11	0.12	0.14	0.15	0.14
Clostridium_perfringens	0.03	0.03	0.05	0.00	0.00	0.00	0.12	0.10	0.10	0.02	0.03	0.03	3.84	3.85	3.72	0.14	0.15	0.18	0.10	0.11	0.11
Clostridium_sp_7_2_43FAA	0.00	0.00	0.00	0.53	0.56	0.51	0.36	0.37	0.36	0.01	0.01	0.01	0.63	0.64	0.60	0.18	0.17	0.17	0.81	0.80	0.81
Clostridium_sp_7_3_54FAA	0.02	0.02	0.02	0.08	0.09	0.09	0.03	0.04	0.03	0.03	0.03	0.03	0.01	0.01	0.01	0.02	0.02	0.02	0.01	0.01	0.01
Clostridium_sp_ATCC_29733	0.00	0.00	0.00	0.10	0.10	0.11	0.07	0.09	0.08	0.05	0.06	0.06	0.07	0.08	0.08	0.02	0.03	0.02	0.08	0.09	0.09
Clostridium_sp_ATCC_BAA_442	0.15	0.17	0.16	0.01	0.01	0.01	0.02	0.03	0.03	0.00	0.01	0.00	0.00	0.00	0.00	0.00	0.00	0.00	0.00	0.00	0.00
Clostridium_sp_D5	0.00	0.00	0.00	0.00	0.00	0.00	0.00	0.00	0.01	0.00	0.00	0.00	0.02	0.02	0.02	0.02	0.01	0.01	0.02	0.02	0.02
Clostridium_sp_M62_1	0.01	0.01	0.02	0.00	0.00	0.00	0.00	0.00	0.00	0.00	0.00	0.00	0.00	0.00	0.00	0.00	0.00	0.00	0.00	0.00	0.00
Clostridium_sp_SS2_1	0.18	0.19	0.15	0.00	0.00	0.00	0.00	0.00	0.00	0.00	0.00	0.00	0.00	0.00	0.00	0.00	0.00	0.00	0.00	0.00	0.00
Clostridium_symbiosum	0.11	0.08	0.10	2.58	2.55	2.57	2.22	2.14	2.14	0.80	0.81	0.79	0.82	0.82	0.84	0.60	0.61	0.57	0.83	0.82	0.86
Parvimonas_unclassified	0.01	0.00	0.00	0.00	0.00	0.00	0.00	0.00	0.00	0.00	0.00	0.00	0.00	0.00	0.00	0.00	0.00	0.00	0.00	0.00	0.00
Clostridiales_bacterium_1_7_47FAA	0.00	0.00	0.00	0.00	0.00	0.00	0.00	0.00	0.00	0.00	0.00	0.00	0.00	0.00	0.00	0.00	0.00	0.00	0.00	0.00	0.00
Flavonifractor_unclassified	0.27	0.29	0.29	0.01	0.01	0.02	0.03	0.03	0.03	0.00	0.01	0.00	0.00	0.00	0.00	0.00	0.00	0.00	0.00	0.00	0.00
Eubacterium_eligens	1.46	1.45	1.44	0.19	0.18	0.19	0.03	0.03	0.03	0.05	0.06	0.05	0.02	0.02	0.02	0.06	0.06	0.06	0.07	0.07	0.07
Eubacterium_hallii	0.01	0.01	0.01	0.00	0.00	0.00	0.00	0.00	0.00	0.00	0.00	0.01	0.00	0.00	0.00	0.00	0.00	0.00	0.02	0.01	0.01
Eubacterium_limosum	0.00	0.00	0.00	0.01	0.01	0.01	0.86	0.86	0.88	0.48	0.48	0.48	0.07	0.07	0.08	0.04	0.04	0.05	0.18	0.19	0.18
Eubacterium_ramulus	0.48	0.47	0.52	0.00	0.00	0.00	0.00	0.00	0.00	0.00	0.00	0.00	0.01	0.01	0.01	0.00	0.00	0.00	0.00	0.00	0.00
Eubacterium_rectale	9.24	9.05	9.31	0.06	0.06	0.06	0.01	0.01	0.01	0.00	0.00	0.00	0.00	0.00	0.00	0.10	0.10	0.11	0.01	0.01	0.01
Eubacterium_siraeum	0.01	0.01	0.01	0.00	0.00	0.00	0.00	0.00	0.00	0.00	0.00	0.00	0.00	0.00	0.00	0.00	0.00	0.00	0.00	0.00	0.00
Eubacterium_ventriosum	0.58	0.55	0.54	0.00	0.00	0.00	0.00	0.00	0.00	0.00	0.00	0.00	0.00	0.00	0.00	0.00	0.00	0.00	0.00	0.00	0.00
Anaerostipes_hadrus	0.13	0.12	0.12	0.00	0.00	0.00	0.00	0.00	0.00	0.00	0.00	0.00	0.00	0.00	0.00	0.00	0.00	0.00	0.00	0.00	0.00
Anaerostipes_sp_3_2_56FAA	0.00	0.00	0.00	0.00	0.00	0.00	0.00	0.00	0.00	0.00	0.00	0.00	0.00	0.00	0.00	0.00	0.00	0.00	0.00	0.00	0.00
Anaerostipes_unclassified	0.00	0.00	0.00	0.01	0.00	0.01	0.00	0.00	0.00	0.00	0.00	0.00	0.02	0.02	0.02	0.00	0.00	0.00	0.00	0.00	0.00
Blautia_producta	0.00	0.00	0.00	0.00	0.00	0.00	0.01	0.00	0.00	0.00	0.00	0.00	0.00	0.00	0.00	0.00	0.00	0.00	0.00	0.00	0.00
Blautia_sp_KLE_1732	0.08	0.09	0.08	0.01	0.00	0.00	0.01	0.01	0.01	0.00	0.00	0.00	0.00	0.00	0.00	0.01	0.01	0.01	0.02	0.02	0.02
Ruminococcus_gnavus	1.70	1.63	1.64	0.08	0.07	0.08	0.05	0.06	0.05	0.34	0.31	0.32	0.02	0.03	0.02	0.07	0.07	0.08	1.53	1.48	1.49
Ruminococcus_obeum	0.36	0.34	0.37	0.01	0.01	0.01	0.01	0.01	0.01	0.02	0.02	0.02	0.00	0.00	0.00	0.09	0.08	0.08	0.09	0.09	0.09
Ruminococcus_torques	1.43	1.40	1.44	0.01	0.02	0.03	0.04	0.03	0.05	0.03	0.04	0.03	0.00	0.00	0.01	0.02	0.02	0.04	0.01	0.00	0.01
Coprococcus_catus	0.10	0.09	0.09	0.01	0.01	0.01	2.47	2.52	2.57	1.41	1.47	1.46	0.06	0.06	0.06	7.21	7.17	7.37	0.82	0.80	0.79

Coprococcus_comes	0.43	0.37	0.38	0.37	0.34	0.39	0.33	0.35	0.33	0.27	0.24	0.25	0.11	0.08	0.09	0.41	0.40	0.38	0.20	0.20	0.19
Coprococcus_sp_HPP0048	0.00	0.00	0.00	0.00	0.00	0.00	0.01	0.01	0.01	0.01	0.00	0.00	0.00	0.00	0.00	0.00	0.00	0.00	0.00	0.00	0.00
Coprococcus_sp_HPP0074	0.00	0.00	0.00	0.00	0.00	0.00	0.00	0.00	0.00	0.00	0.00	0.00	0.00	0.00	0.00	0.00	0.00	0.00	0.00	0.00	0.00
Dorea_formicigenerans	1.90	1.93	1.89	0.88	0.89	0.89	0.90	0.90	0.86	0.95	0.88	0.90	0.46	0.46	0.45	0.48	0.50	0.47	0.43	0.41	0.40
Dorea_longicatena	1.87	1.84	1.90	0.03	0.04	0.03	0.09	0.10	0.09	0.05	0.06	0.07	0.03	0.04	0.04	0.08	0.09	0.10	0.03	0.04	0.04
Lachnospiraceae_bacterium_1_1_57FAA	0.24	0.24	0.17	0.00	0.00	0.00	0.03	0.01	0.02	0.01	0.00	0.00	0.00	0.00	0.00	0.00	0.01	0.00	0.00	0.00	0.00
Lachnospiraceae_bacterium_1_4_56FAA	0.01	0.01	0.01	0.00	0.00	0.00	0.00	0.00	0.00	0.00	0.00	0.00	0.00	0.01	0.01	0.00	0.00	0.00	0.00	0.00	0.00
Lachnospiraceae_bacterium_2_1_58FAA	0.04	0.05	0.03	0.00	0.00	0.00	0.00	0.00	0.00	0.00	0.01	0.00	0.00	0.00	0.00	0.00	0.00	0.00	0.01	0.01	0.01
Lachnospiraceae_bacterium_3_1_46FAA	0.07	0.08	0.07	0.00	0.00	0.00	0.00	0.00	0.00	0.00	0.00	0.00	0.00	0.00	0.00	0.00	0.00	0.00	0.00	0.00	0.00
Lachnospiraceae_bacterium_3_1_57FAA_CT1	0.00	0.00	0.00	0.00	0.00	0.00	0.00	0.00	0.00	0.00	0.00	0.00	0.00	0.00	0.00	0.16	0.18	0.17	0.01	0.01	0.01
Lachnospiraceae_bacterium_4_1_37FAA	0.00	0.00	0.00	0.00	0.00	0.00	0.01	0.01	0.00	0.00	0.00	0.00	0.00	0.00	0.00	0.00	0.00	0.00	0.00	0.00	0.00
Lachnospiraceae_bacterium_5_1_57FAA	0.00	0.00	0.00	0.00	0.00	0.00	0.01	0.01	0.01	0.01	0.00	0.01	0.00	0.00	0.00	0.00	0.00	0.00	0.01	0.01	0.02
Lachnospiraceae_bacterium_5_1_63FAA	0.16	0.19	0.18	0.00	0.00	0.00	0.00	0.00	0.00	0.00	0.00	0.00	0.00	0.00	0.00	0.00	0.00	0.00	0.00	0.00	0.00
Lachnospiraceae_bacterium_7_1_58FAA	0.05	0.05	0.05	0.00	0.00	0.00	0.01	0.01	0.01	0.00	0.00	0.00	0.00	0.00	0.00	0.00	0.00	0.00	0.00	0.00	0.00
Lachnospiraceae_bacterium_8_1_57FAA	0.44	0.46	0.72	0.01	0.00	0.00	0.01	0.01	0.03	0.00	0.03	0.00	0.00	0.00	0.00	0.00	0.00	0.00	0.00	0.01	0.00
Lachnospiraceae_bacterium_9_1_43BFAA	0.00	0.00	0.00	0.00	0.00	0.00	0.00	0.00	0.00	0.00	0.00	0.00	0.00	0.00	0.00	0.00	0.00	0.00	0.00	0.00	0.00
Roseburia_hominis	0.13	0.14	0.13	0.00	0.00	0.00	0.00	0.00	0.00	0.00	0.00	0.00	0.00	0.00	0.00	0.00	0.00	0.00	0.00	0.00	0.00
Roseburia_intestinalis	0.92	0.76	0.77	0.00	0.00	0.00	0.00	0.00	0.00	0.00	0.00	0.00	0.00	0.00	0.00	0.00	0.00	0.00	0.00	0.00	0.00
Roseburia_inulinivorans	1.01	0.95	0.92	0.00	0.00	0.00	0.00	0.00	0.00	0.00	0.00	0.00	0.00	0.00	0.00	0.01	0.00	0.00	0.00	0.00	0.00
Roseburia_unclassified	0.00	0.00	0.00	0.00	0.00	0.00	0.00	0.00	0.00	0.00	0.00	0.00	0.00	0.00	0.00	0.00	0.00	0.01	0.00	0.00	0.00
Oscillibacter_sp_KLE_1728	0.06	0.05	0.08	0.00	0.00	0.00	0.00	0.00	0.00	0.00	0.00	0.00	0.00	0.00	0.00	0.00	0.00	0.00	0.00	0.00	0.00
Oscillibacter_sp_KLE_1745	0.02	0.02	0.02	0.00	0.00	0.00	0.00	0.00	0.00	0.00	0.00	0.00	0.00	0.00	0.00	0.00	0.00	0.00	0.00	0.00	0.00
Clostridium_bartlettii	0.01	0.01	0.01	0.00	0.00	0.00	0.00	0.00	0.00	0.00	0.00	0.00	0.00	0.00	0.00	0.00	0.00	0.00	0.00	0.00	0.00
Clostridium_bifermentans	0.00	0.00	0.00	0.00	0.00	0.00	0.02	0.02	0.01	0.00	0.00	0.00	0.00	0.00	0.00	0.01	0.01	0.01	0.05	0.05	0.04
Clostridium_difficile	0.00	0.00	0.00	0.03	0.04	0.04	0.05	0.05	0.05	0.07	0.06	0.06	0.13	0.12	0.12	0.06	0.05	0.07	0.02	0.01	0.02
Clostridium_sordellii	0.00	0.00	0.00	0.03	0.03	0.03	0.00	0.00	0.00	0.00	0.00	0.00	0.00	0.00	0.00	0.00	0.01	0.01	0.00	0.00	0.00
Peptostreptococcaceae_noname_unclassified	0.00	0.00	0.00	0.15	0.09	0.08	0.00	0.00	0.01	0.00	0.00	0.04	0.06	0.00	0.00	0.07	0.10	0.10	0.02	0.00	0.03
Anaerotruncus_colihominis	0.03	0.03	0.04	0.00	0.00	0.00	0.00	0.00	0.00	0.00	0.00	0.00	0.00	0.00	0.00	0.00	0.00	0.00	0.00	0.00	0.00
Anaerotruncus_unclassified	0.02	0.00	0.00	0.00	0.01	0.00	0.00	0.00	0.00	0.00	0.00	0.00	0.00	0.00	0.00	0.00	0.00	0.00	0.00	0.00	0.00
Faecalibacterium_prausnitzii	5.38	5.32	5.59	0.00	0.00	0.00	0.00	0.00	0.00	0.00	0.00	0.00	0.00	0.00	0.00	0.00	0.00	0.00	0.00	0.00	0.00
Faecalibacterium_unclassified	6.49	7.17	6.94	0.08	0.09	0.09	0.01	0.01	0.01	0.01	0.01	0.01	0.02	0.02	0.02	0.02	0.02	0.02	0.05	0.07	0.06
Ruminococcaceae_bacterium_D16	0.10	0.11	0.11	0.01	0.00	0.01	0.00	0.00	0.00	0.00	0.00	0.00	0.00	0.00	0.00	0.00	0.00	0.00	0.00	0.00	0.00
Ruminococcus_bromii	0.07	0.07	0.06	0.00	0.00	0.00	0.00	0.00	0.00	0.00	0.00	0.00	0.00	0.00	0.00	0.01	0.01	0.00	0.00	0.00	0.00
Ruminococcus_lactaris	1.64	1.64	1.65	0.03	0.03	0.03	0.01	0.01	0.01	0.01	0.01	0.01	0.01	0.00	0.01	0.01	0.02	0.01	0.01	0.01	0.01
Ruminococcus_sp	0.08	0.08	0.07	0.00	0.00	0.00	0.00	0.00	0.00	0.00	0.00	0.00	0.00	0.00	0.00	0.00	0.00	0.00	0.01	0.00	0.00
Ruminococcus_sp_5_1_39BFAA	0.77	0.78	0.79	0.09	0.08	0.08	0.00	0.00	0.01	0.03	0.03	0.04	0.00	0.00	0.00	0.16	0.18	0.22	0.18	0.18	0.16
Ruminococcus_sp_JC304	0.01	0.01	0.01	0.00	0.00	0.00	0.00	0.00	0.00	0.00	0.00	0.00	0.00	0.00	0.00	0.00	0.00	0.00	0.00	0.00	0.00
Subdoligranulum_sp_4_3_54A2FAA	0.09	0.11	0.10	0.06	0.07	0.07	0.07	0.09	0.08	0.07	0.07	0.07	0.06	0.07	0.07	0.05	0.04	0.04	0.11	0.13	0.13
Subdoligranulum_unclassified	4.21	4.20	3.84	0.34	0.31	0.23	0.68	0.66	0.66	0.74	0.66	0.76	0.29	0.24	0.26	0.68	0.62	0.51	0.14	0.13	0.12
Catenibacterium_unclassified	0.01	0.02	0.01	0.00	0.00	0.00	0.04	0.04	0.05	0.39	0.41	0.39	0.00	0.00	0.00	0.05	0.05	0.06	0.01	0.01	0.01
Coprobacillus_sp_3_3_56FAA	0.00	0.00	0.00	0.00	0.00	0.00	0.00	0.00	0.00	0.02	0.01	0.01	0.00	0.00	0.00	0.01	0.01	0.01	0.04	0.05	0.04
Coprobacillus_sp_8_2_54BFAA	0.00	0.00	0.00	0.00	0.00	0.00	0.00	0.00	0.00	0.00	0.01	0.00	0.00	0.00	0.00	0.00	0.00	0.00	0.03	0.02	0.02
Coprobacillus_sp_D7	0.00	0.00	0.00	0.00	0.00	0.00	0.00	0.00	0.00	0.03	0.03	0.04	0.00	0.00	0.00	0.03	0.02	0.02	0.10	0.09	0.12
Coprobacillus_unclassified	0.00	0.00	0.00	0.02	0.02	0.02	0.03	0.03	0.03	0.33	0.33	0.32	0.03	0.03	0.03	0.16	0.16	0.18	0.90	0.89	0.85
Clostridium_ramosum	0.00	0.00	0.00	0.00	0.00	0.00	0.01	0.00	0.00	0.05	0.05	0.05	0.00	0.00	0.00	0.04	0.04	0.03	0.22	0.25	0.21
Clostridium_spiroforme	0.01	0.01	0.01	0.00	0.00	0.00	0.00	0.00	0.00	0.00	0.00	0.00	0.00	0.00	0.00	0.00	0.00	0.00	0.00	0.00	0.00
Erysipelotrichaceae_bacterium_2_2_44A	0.01	0.00	0.00	0.00	0.00	0.00	0.00	0.00	0.00	0.00	0.00	0.00	0.00	0.00	0.00	0.00	0.00	0.00	0.00	0.00	0.00
Eubacterium_biforme	0.69	0.65	0.61	0.10	0.09	0.10	0.05	0.05	0.05	0.20	0.19	0.18	0.04	0.04	0.03	0.07	0.07	0.08	0.10	0.10	0.09
Eubacterium_dolichum	0.00	0.00	0.01	0.00	0.00	0.00	0.00	0.00	0.00	0.00	0.00	0.00	0.00	0.00	0.00	0.00	0.00	0.00	0.00	0.00	0.00
Holdemania_filiformis	0.04	0.04	0.03	0.07	0.08	0.09	0.08	0.09	0.10	0.03	0.02	0.02	0.01	0.01	0.02	0.02	0.03	0.02	0.03	0.03	0.04
Holdemania_sp_AP2	0.04	0.04	0.04	0.02	0.02	0.02	0.02	0.02	0.03	0.00	0.00	0.01	0.00	0.00	0.00	0.01	0.01	0.01	0.00	0.00	0.01
Holdemania_unclassified	0.00	0.00	0.00	0.00	0.00	0.00	0.00	0.00	0.00	0.00	0.00	0.00	0.00	0.00	0.00	0.00	0.00	0.00	0.00	0.00	0.00
Phascolarctobacterium_unclassified	0.95	0.87	0.93	0.54	0.54	0.55	0.18	0.19	0.16	0.07	0.08	0.08	0.01	0.02	0.02	0.30	0.31	0.33	0.23	0.22	0.22
Dialister_invisus	3.62	3.44	3.60	0.02	0.02	0.01	0.00	0.00	0.00	0.00	0.00	0.00	0.00	0.00	0.00	0.00	0.00	0.00	0.00	0.00	0.00
Dialister_succinatiphilus	0.02	0.02	0.03	0.00	0.00	0.00	0.00	0.00	0.00	0.00	0.00	0.00	0.00	0.00	0.00	0.00	0.00	0.00	0.00	0.00	0.00
Megamonas_funiformis	0.85	0.75	0.83	0.09	0.09	0.10	0.04	0.03	0.03	0.05	0.06	0.06	0.09	0.08	0.09	0.32	0.29	0.35	0.11	0.12	0.11
Megamonas_hypermegale	0.57	0.59	0.56	0.07	0.06	0.06	0.02	0.02	0.02	0.05	0.03	0.04	0.07	0.07	0.07	0.18	0.21	0.20	0.06	0.06	0.06
Megamonas_rupellensis	0.13	0.12	0.11	0.00	0.00	0.00	0.00	0.00	0.00	0.00	0.01	0.00	0.01	0.01	0.01	0.02	0.02	0.02	0.00	0.00	0.00
Megamonas_unclassified	6.42	6.41	6.33	0.74	0.73	0.74	0.27	0.27	0.28	0.51	0.48	0.46	0.83	0.83	0.76	2.27	2.28	2.38	0.83	0.78	0.80
Veillonella_atypica	0.02	0.03	0.02	0.00	0.00	0.00	0.21	0.20	0.21	0.58	0.56	0.57	0.00	0.00	0.00	0.88	0.88	0.88	0.00	0.00	0.00
Veillonella_dispar	0.01	0.01	0.01	0.00	0.00	0.00	0.16	0.16	0.14	0.22	0.20	0.20	0.02	0.02	0.02	0.19	0.18	0.20	0.03	0.02	0.02
Veillonella_parvula	0.05	0.05	0.05	0.01	0.01	0.01	0.81	0.78	0.73	0.75	0.77	0.75	0.10	0.10	0.10	0.51	0.51	0.49	0.35	0.34	0.33
Veillonella_sp_3_1_44	0.00	0.00	0.00	0.00	0.00	0.00	0.04	0.04	0.02	0.08	0.06	0.06	0.00	0.00	0.00	0.03	0.03	0.04	0.01	0.00	0.00
Veillonella_sp_6_1_27	0.00	0.00	0.00	0.00	0.00	0.00	0.00	0.00	0.01	0.01	0.01	0.02	0.00	0.00	0.00	0.01	0.01	0.00	0.00	0.01	0.00
Veillonella_sp_ACP1	0.00	0.00	0.00	0.00	0.00	0.00	0.00	0.00	0.00	0.01	0.01	0.01	0.00	0.00	0.00	0.00	0.00	0.00	0.00	0.00	0.00
Veillonella_sp_HPA0037	0.00	0.00	0.00	0.00	0.00	0.00	0.02	0.01	0.02	0.03	0.03	0.05	0.00	0.00	0.00	0.08	0.07	0.07	0.00	0.00	0.00
Veillonella_sp_oral_taxon_158	0.02	0.01	0.01	0.00	0.00	0.00	0.15	0.15	0.14	0.08	0.08	0.07	0.01	0.01	0.01	0.08	0.08	0.07	0.01	0.02	0.01
Veillonella_unclassified	0.04	0.05	0.02	0.01	0.01	0.01	0.25	0.28	0.30	0.42	0.33	0.34	0.11	0.10	0.09	0.19	0.20	0.22	0.09	0.08	0.12
Fusobacterium_nucleatum	0.00	0.00	0.00	0.00	0.00	0.00	0.00	0.00	0.00	0.00	0.00	0.00	0.00	0.00	0.00	0.00	0.00	0.00	0.00	0.00	0.00
Fusobacterium_ulcerans	0.00	0.00	0.00	0.00	0.00	0.00	0.00	0.00	0.00	0.00	0.00	0.00	0.00	0.00	0.00	0.00	0.00	0.00	0.00	0.00	0.00
Fusobacterium_varium	0.00	0.00	0.00	5.64	5.35	5.35	7.54	7.34	7.30	5.66	5.41	5.42	3.90	3.45	3.48	5.81	5.49	5.82	6.85	6.48	6.76
Burkholderiales_bacterium_1_1_47	0.01	0.01	0.01	0.01	0.01	0.01	0.00	0.00	0.00	0.00	0.00	0.00	0.00	0.00	0.00	0.00	0.00	0.00	0.00	0.00	0.00
Parasutterella_unclassified	0.00	0.01	0.00	0.00	0.01	0.01	0.00	0.00	0.00	0.00	0.00	0.00	0.00	0.00	0.00	0.00	0.00	0.00	0.00	0.00	0.00

Sutterella_wadsworthensis	0.46	0.47	0.46	0.26	0.26	0.27	1.40	1.48	1.47	0.96	0.99	0.99	0.32	0.29	0.27	0.51	0.51	0.52	0.83	0.84	0.73
Bilophila_sp_4_1_30	0.07	0.09	0.08	0.89	0.87	0.92	0.44	0.46	0.45	0.26	0.25	0.26	0.17	0.17	0.18	0.24	0.26	0.26	0.24	0.26	0.24
Bilophila_unclassified	0.82	0.81	0.85	8.26	8.88	8.96	3.96	4.31	4.25	2.30	2.63	2.52	1.56	1.68	1.66	2.36	2.52	2.39	2.23	2.46	2.42
Bilophila_wadsworthia	0.04	0.05	0.05	0.40	0.37	0.43	0.19	0.20	0.21	0.10	0.11	0.11	0.08	0.07	0.08	0.11	0.12	0.12	0.10	0.10	0.10
Desulfovibrio_piger	0.00	0.00	0.00	0.08	0.10	0.12	0.03	0.03	0.03	0.02	0.02	0.02	0.03	0.02	0.02	0.09	0.09	0.08	0.02	0.04	0.03
Citrobacter_freundii	0.02	0.02	0.02	0.56	0.56	0.57	0.20	0.21	0.21	0.19	0.19	0.18	0.34	0.32	0.33	0.19	0.17	0.17	0.76	0.72	0.72
Citrobacter_sp_30_2	0.00	0.00	0.00	0.01	0.01	0.00	0.00	0.00	0.00	0.01	0.00	0.00	0.01	0.00	0.01	0.01	0.01	0.01	0.02	0.02	0.01
Citrobacter_sp_KTE151	0.00	0.00	0.00	0.00	0.00	0.00	0.00	0.00	0.00	0.00	0.00	0.00	0.00	0.00	0.00	0.00	0.00	0.00	0.00	0.00	0.01
Citrobacter_sp_KTE30	0.00	0.00	0.00	0.00	0.00	0.00	0.00	0.00	0.00	0.00	0.00	0.00	0.00	0.00	0.00	0.00	0.00	0.00	0.01	0.00	0.00
Citrobacter_unclassified	0.03	0.03	0.03	0.55	0.54	0.57	0.14	0.14	0.14	0.11	0.12	0.12	0.25	0.26	0.30	0.08	0.11	0.12	0.52	0.64	0.62
Enterobacter_aerogenes	0.01	0.01	0.01	0.14	0.14	0.15	0.00	0.00	0.00	0.03	0.03	0.03	0.03	0.03	0.03	0.06	0.07	0.06	0.04	0.05	0.05
Enterobacter_cloacae	0.01	0.02	0.01	0.14	0.13	0.13	0.12	0.14	0.14	0.08	0.08	0.11	0.13	0.12	0.12	0.27	0.25	0.28	0.22	0.27	0.25
Escherichia_coli	1.25	0.93	1.24	35.97	35.31	35.51	14.64	14.29	13.51	17.69	17.92	17.05	32.03	29.94	30.15	18.43	18.96	20.41	13.45	14.34	13.58
Escherichia_unclassified	0.00	0.72	0.24	10.06	11.64	10.91	3.37	4.02	4.56	3.42	4.85	5.27	6.01	9.90	9.63	5.25	5.21	3.51	4.04	4.14	4.64
Hafnia_unclassified	0.00	0.00	0.00	0.01	0.01	0.01	0.00	0.00	0.00	0.00	0.00	0.00	0.00	0.00	0.00	0.00	0.00	0.00	0.00	0.01	0.01
Klebsiella_oxytoca	0.00	0.00	0.00	0.04	0.04	0.04	0.00	0.00	0.01	0.03	0.03	0.03	0.05	0.05	0.05	0.10	0.10	0.10	0.05	0.05	0.05
Klebsiella_pneumoniae	0.02	0.04	0.02	0.30	0.27	0.20	0.16	0.19	0.13	0.14	0.19	0.23	0.50	0.72	0.51	0.77	0.97	0.95	0.50	0.50	0.53
Klebsiella_unclassified	0.00	0.00	0.00	0.00	0.00	0.03	0.00	0.00	0.02	0.04	0.00	0.00	0.04	0.00	0.24	0.00	0.00	0.00	0.07	0.03	0.04
Raoultella_unclassified	0.00	0.00	0.00	0.00	0.00	0.00	0.00	0.00	0.00	0.00	0.00	0.00	0.00	0.00	0.00	0.00	0.00	0.00	0.00	0.00	0.00
Haemophilus_parainfluenzae	0.09	0.08	0.10	0.00	0.00	0.00	0.00	0.00	0.00	0.00	0.00	0.00	0.00	0.00	0.00	0.00	0.00	0.00	0.00	0.00	0.00
Synergistes_unclassified	0.01	0.01	0.01	0.00	0.00	0.00	0.00	0.00	0.00	0.00	0.00	0.00	0.00	0.00	0.00	0.00	0.00	0.00	0.00	0.00	0.00
Akkermansia_unclassified	0.00	0.00	0.00	0.00	0.00	0.00	0.00	0.00	0.00	0.00	0.00	0.00	0.00	0.00	0.00	0.16	0.16	0.17	0.01	0.01	0.01
