# Supplementary material for: Evaluating the prebiotic effect of oligosaccharides on gut microbiome wellness using in vitro fecal fermentation
Source: NPJ Sci Food. 2023 May 9;7:18. doi: 10.1038/s41538-023-00195-1 (PMC10170090; doi:10.1038/s41538-023-00195-1)
Supplement: Supplementary file 2 — Reporting Summary [file 41538_2023_195_MOESM2_ESM.pdf]

nature portfolio

Corresponding author(s): Nam Soo Han

Last updated by author(s): Apr 14, 2023

Reporting Summary

Nature Portfolio wishes to improve the reproducibility of the work that we publish. This form provides structure for consistency and transparency in reporting. For further information on Nature Portfolio policies, see our Editorial Policies and the Editorial Policy Checklist.

Statistics

For all statistical analyses, confirm that the following items are present in the figure legend, table legend, main text, or Methods section.

n/a	Confirmed	
☐	☒	The exact sample size (*n*) for each experimental group/condition, given as a discrete number and unit of measurement
☐	☒	A statement on whether measurements were taken from distinct samples or whether the same sample was measured repeatedly
☐	☒	The statistical test(s) used AND whether they are one- or two-sided<br>*Only common tests should be described solely by name; describe more complex techniques in the Methods section.*
☐	☒	A description of all covariates tested
☐	☒	A description of any assumptions or corrections, such as tests of normality and adjustment for multiple comparisons
☐	☒	A full description of the statistical parameters including central tendency (e.g. means) or other basic estimates (e.g. regression coefficient) AND variation (e.g. standard deviation) or associated estimates of uncertainty (e.g. confidence intervals)
☐	☒	For null hypothesis testing, the test statistic (e.g. *F*, *t*, *r*) with confidence intervals, effect sizes, degrees of freedom and *P* value noted<br>*Give P values as exact values whenever suitable.*
☒	☐	For Bayesian analysis, information on the choice of priors and Markov chain Monte Carlo settings
☒	☐	For hierarchical and complex designs, identification of the appropriate level for tests and full reporting of outcomes
☒	☐	Estimates of effect sizes (e.g. Cohen's *d*, Pearson's *r*), indicating how they were calculated

Our web collection on statistics for biologists contains articles on many of the points above.

Software and code

Policy information about availability of computer code

Data collection	No software was used to collect the data in study.
Data analysis	Line 295-298: Sequence reads were processed with the KneadData v0.5.1 quality-control pipeline (http://huttenhower.sph.harvard.edu/kneaddata), which uses Trimmomatic v0.39 and Bowtie2 v0.1, to remove low-quality read bases and human reads, respectively.<br>Line 302-303: Taxonomic profiling was conducted using the MetaPhlAn2 v2.7.0 taxonomic sequence classification system with default parameters.<br>Line 306-308: The Gut Microbiome Wellness Index (GMWI) was calculated using GMWI-webtool, a user-friendly browser application that computes GMWI from shotgun metagenome taxonomic profiles of stool.

For manuscripts utilizing custom algorithms or software that are central to the research but not yet described in published literature, software must be made available to editors and reviewers. We strongly encourage code deposition in a community repository (e.g. GitHub). See the Nature Portfolio guidelines for submitting code & software for further information.

Data

Policy information about availability of data

All manuscripts must include a data availability statement. This statement should provide the following information, where applicable:

- Accession codes, unique identifiers, or web links for publicly available datasets
- A description of any restrictions on data availability
- For clinical datasets or third party data, please ensure that the statement adheres to our policy

The raw sequence reads obtained for this study are deposited at https://www.ncbi.nlm.nih.gov/sra/PRJNA902022.&#x201D and can be accessed without restrictions. The remaining data from the current study are available in the main text or in the supplementary information.

Human research participants

Policy information about studies involving human research participants and Sex and Gender in Research.

Reporting on sex and gender	Line 273-276: Fecal samples were obtained from 19 healthy adult volunteers (14 males and 5 females, age from 25 to 30 years) who were not consuming antibiotics or pre/probiotics at the time of the study, and had no recent history of gastrointestinal disorders.
Population characteristics	No data was included from the human.
Recruitment	No data was included from the human.
Ethics oversight	No data was included from the human.

Note that full information on the approval of the study protocol must also be provided in the manuscript.

Field-specific reporting

Please select the one below that is the best fit for your research. If you are not sure, read the appropriate sections before making your selection.

☒ Life sciences ☐ Behavioural & social sciences ☐ Ecological, evolutionary & environmental sciences

For a reference copy of the document with all sections, see nature.com/documents/nr-reporting-summary-flat.pdf

Life sciences study design

All studies must disclose on these points even when the disclosure is negative.

Sample size	No sample size calculations were performed. The sample size (n) of each experiment is provided in the statistical analysis section in the main manuscript. Sample sizes were chosen to support meaningful conclusions.
Data exclusions	No data was excluded from the analyses.
Replication	All in vitro experiments were replicated 3 times.
Randomization	In this study, each group consisted of identically engineered samples. The work does not involve participant groups. Therefore, randomization was not relevant the study.
Blinding	The work does not involve participant groups. Therefore, blinding was not relevant to the study.

Reporting for specific materials, systems and methods

We require information from authors about some types of materials, experimental systems and methods used in many studies. Here, indicate whether each material, system or method listed is relevant to your study. If you are not sure if a list item applies to your research, read the appropriate section before selecting a response.

Materials & experimental systems

n/a	Involved in the study	
☒	☐	Antibodies
☒	☐	Eukaryotic cell lines
☒	☐	Palaeontology and archaeology
☒	☐	Animals and other organisms
☒	☐	Clinical data
☒	☐	Dual use research of concern

Methods

n/a	Involved in the study	
☒	☐	ChIP-seq
☒	☐	Flow cytometry
☒	☐	MRI-based neuroimaging
